# Prospective Physical Activity, Sitting and Sleep consortium (ProPASS): addressing methodological and geographical barriers to inform global public health guidelines, interventions and precision medicine

**DOI:** 10.1136/bjsports-2025-111283

**Published:** 2026-06-22

**Authors:** Emmanuel Stamatakis, Nicholas A Koemel, John J Mitchell, Andrew J Atkin, Richard Pulsford, Kar Hau Chong, Vegar Rangul, Joanna M Blodgett, Raaj Kishore Biswas, Carlos A Celis-Morales, Lauren Sherar, Gita Mishra, Joanne McVeigh, Marla K Beauchamp, Sebastian Jannas-Vela, Christina Bjørk Petersen, Mette Korshøj, Mette Aadahl, Randi Jepsen, Laura Karavirta, Sari Stenholm, Séverine Sabia, David Richter, Peter A Cistulli, Timothy Chi-yui Kwok, Yuta Nemoto, Dick H J Thijssen, Thijs Eijsvogels, Paul Jarle Mork, Abdulrahman I Alaqil, Falk Müller-Riemenschneider, Lisa Micklesfield, Katia Gallegos-Carrillo, Gianluca Severi, Parminder Raina, Verónica Cabanas-Sánchez, Amal Wanigatunga, Eleanor M Simonsick, Jennifer A Schrack, Jesús del Pozo-Cruz, Esmée A Bakker, Andreas Holtermann, I-Min Lee, Borja del Pozo Cruz, Annemarie Koster, Matthew N Ahmadi, Mark Hamer, ProPASS Collaboration, Pasan Hettiarachchi

**Affiliations:** 1 Turner Institute for Brain and Mental Health, School of Psychological Sciences, Faculty of Medicine, Nursing and Health Sciences, Monash University, Melbourne, Victoria, Australia; 2 Mackenzie Wearables Research Hub, Charles Perkins Centre, The University of Sydney, Sydney, New South Wales, Australia; 3 Victorian Heart Institute, Monash University, Melbourne, Victoria, Australia; 4 University College London Hospitals, National Institute for Health and Care Research Biomedical Research Centre, London, UK; 5 School of Health Sciences and Norwich Epidemiology Centre, University of East Anglia, Norwich, UK; 6 Faculty of Health and Life Sciences, University of Exeter, Exeter, UK; 7 HUNT Research Centre, Department of Public Health and Nursing, Faculty of Medicine and Health Sciences, Norwegian University of Science and Technology, Trondheim, Norway; 8 Institute of Sport, Exercise and Health, Division of Surgery and Interventional Sciences, University College London, London, UK; 9 School of Cardiovascular and Metabolic Health, University of Glasgow, Glasgow, UK; 10 Human Performance Lab Education, Physical Activity, and Health Research Unit, Universidad Católica del Maule, Talca, Chile; 11 School of Sport, Exercise and Health Sciences, Loughborough University, Loughborough, UK; 12 School of Public Health, University of Queensland, Brisbane, Queensland, Australia; 13 Curtin School of Allied Health, Curtin University, Perth, Western Australia, Australia; 14 School of Rehabilitation Science, McMaster University, Hamilton, Ontario, Canada; 15 Universidad de O’Higgins, Instituto de Ciencias de la Salud, Rancagua, Chile; 16 National Institute of Public Health, University of Southern Denmark, Odense, Denmark; 17 Department of Occupational and Social Medicine, Copenhagen University Hospital Holbæk, Copenhagen, Denmark; 18 Center for Clinical Research and Prevention, Bispebjerg and Frederiksberg Hospital, Frederiksberg, Denmark; 19 Centre of Health Research, Zealand University Hospital Nykøbing F, Nykøbing Falster, Region Zealand, Denmark; 20 Gerontology Research Center and Faculty of Sport and Health Sciences, University of Jyväskylä, Jyväskylä, Central Finland, Finland; 21 Department of Public Health, University of Turku and Turku University Hospital, Turku, Finland; 22 Inserm U1153, CRESS, EpiAgeing, Université Paris Cité, Paris, France; 23 SHARE Berlin Institute, Berlin, Germany; 24 Charles Perkins Centre, Faculty of Medicine and Health, The University of Sydney, Sydney, New South Wales, Australia; 25 Department of Medicine and Therapeutics, Faculty of Medicine, The Chinese University of Hong Kong, Hong Kong, People's Republic of China; 26 School of Nursing, The Hong Kong Polytechnic University, Hong Kong, People's Republic of China; 27 School of Health Innovation, Kanagawa University of Human Services, Kanagawa, Kanagawa Prefecture, Japan; 28 Department of Medical BioSciences, Radboud University Medical Center, Nijmegen, Gelderland, Netherlands; 29 Department of Physical Education, College of Education, King Faisal University, Al Ahsa, Eastern Province, Saudi Arabia; 30 Saw Swee Hock School of Public Health, National University of Singapore, Singapore; 31 SAMRC/Wits Developmental Pathways for Health Research Unit, Department of Paediatrics, School of Clinical Medicine, University of the Witwatersrand Faculty of Health Sciences, Johannesburg, Gauteng, South Africa; 32 Epidemiology and Health Services Research Unit, Mexican Institute of Social Security, Morelos, Mexico; 33 UVSQ, Inserm U1018 CESP, Université Paris-Saclay, Paris, France; 34 McMaster Institute for Research on Aging and Department of Health Evidence, Impact and Evidence, Faculty of Health Sciences, McMaster University, Hamilton, Ontario, Canada; 35 Department of Preventive Medicine and Public Health, School of Medicine, Universidad Autonoma de Madrid, Madrid, Spain; 36 CIBER of Epidemiology and Public Health, Madrid, Community of Madrid, Spain; 37 Department of Epidemiology, Johns Hopkins University Bloomberg School of Public Health, Baltimore, Maryland, USA; 38 National Institute on Aging, Bethesda, Maryland, USA; 39 University of Seville, Seville, Spain; 40 National Research Centre for the Working Environment, Copenhagen, Denmark; 41 Division of Preventive Medicine, Brigham and Women’s Hospital and Harvard Medical School, Boston, Massachusetts, USA; 42 Department of Sport Sciences, Faculty of Medicine, Health and Sports, Universidad Europea de Madrid Campus de Villaviciosa de Odón, Madrid, Spain; 43 CAPRHI Care and Public Health Research Institute, Department of Social Medicine, Maastricht University, Maastricht, Netherlands

**Keywords:** Public health, Epidemiology, Sleep, Physical activity

## Background

 To date, public health guidance on physical activity, sedentary behaviour and sleep has been primarily informed by studies that relied on self-report methods (mostly recall questionnaires) to characterise and quantify behaviour. This class of evidence has established physical inactivity as an important risk factor for non-communicable diseases that is served by guidelines, interventions and policy. However, it is widely recognised that self-reported data suffer from numerous limitations, including recall and social desirability biases as well as poor validity and precision. These limitations can lead to an underestimation of effect sizes. In addition, questionnaires on physical activity and sedentary behaviour restrict data coverage to limited parts of the 24-hour day, typically to structured and planned activities. Self-reported sleep information also suffers from low-resolution, often reported in hourly increments with no indication of sleep quality or timing. These limitations reduce the utility of such data for characterising the full 24-hour profile for integrated holistic behavioural interventions and precision medicine. Wearable devices, such as accelerometers, represent a significant leap forward as they overcome many of these limitations. Despite wearables being introduced in cohort studies over 20 years ago,^1^ their contribution to public health guidelines has been very limited to date. For example, such research contributed only a small fraction (estimated to be 5–10%) of the evidence synthesised in the WHO 2020 Physical Activity and Sedentary Behaviour Guidelines,^2^ prompting the Guidelines Development Group to flag the need for a gradual transition from self-report to wearables-based guidelines.^3^ The surprisingly small contribution of wearables-based evidence to guidelines is, in part, due to methodological heterogeneity within the evidence base and the slow progress with developing universal standards. Except for two children’s consortia, the International Children’s Accelerometry Database and the Sleep and Activity Database for the Early Years, which were limited to devices worn on the waist,^4 5^ no other epidemiological consortium of studies with wearables data existed until recently.

The transition from self-report to device-based assessments has yielded substantial advances in intervention-relevant knowledge and guidelines that were previously unattainable. For example, accelerometry has enabled the discovery of physical activity micropatterns, defined as brief intermittent bursts of vigorous or moderate-to-vigorous physical activity, that are consistently associated with substantially lower mortality and morbidity risks.^6 7^ These micropatterns cannot be identified by questionnaires.^8^ Device-measured data have also revealed precise non-linear dose–response relationships, including specific thresholds for sedentary time^9^ and equivalence of intensities.^10^ Devices providing continuous 24-hour measurement have enabled compositional analyses demonstrating that the health impact of increasing physical activity depends critically on which behaviour (sleep, sedentary time or light activity) is displaced,^11^ information that is not possible to obtain from questionnaires assessing behaviours in isolation. Additional examples include the identification of sleep irregularity and fragmentation patterns as cardiometabolic risk factors^12 13^ and as determinants of mortality^14^, independent of duration.

## Overview of Prospective Physical Activity, Sitting and Sleep consortium

Established in 2017–2018, the Prospective Physical Activity, Sitting and Sleep consortium (ProPASS)^15–18^ is a large international collaboration of cohort studies with research-grade wearables data aimed at advancing our understanding of the associations of free-living physical activity, posture (sitting, standing) and sleep with major health and non-communicable disease outcomes. From inception, ProPASS’ overarching goal has been to spearhead the transition from self-report to wearables-based evidence for guidelines and evidence to inform more targeted behaviour change interventions.^19^ To achieve this goal, ProPASS aims to compile a number of data resources and make them available to the global research community. Two of ProPASS’ unique features include a parallel focus on recruiting cohorts: (a) retrospectively (ie, inclusion of cohorts with existing wearables data), and (b) prospectively (ie, support cohorts without wearables data to carry out such data collection and join ProPASS). A key step is the pooling of these data sources to create a single data resource. New cohort recruitment is very resource-demanding, but offers key advantages, including prospective harmonisation of contextual data such as socioeconomic status. Another unique aspect of ProPASS is its strong concurrent focus on developing and refining wearables methodologies,^20 21^ including algorithm and software development,^22–29^ statistical and causal inference methods, fieldwork protocols, and at-scale harmonisation workflows. ProPASS currently has specific work streams that are presented in detail in table 1 and online supplemental figure 1, and include technical (eg, statistics, wearable measurement, federated data infrastructure and PASS*omni* (formerly known as ActiPASS) software development (wearables data processing software)), data linkage, dissemination-related (scientific outputs) and strategic (eg, consortium expansion group and the new Clinical Physical Activity, Sedentary Behaviour and Sleep consortium (ClinPASS) arm) streams.

**Table 1:** ProPASS working group streams

ProPASS stream	Aim
Harmonisation	Leading the standardisation of data collection, processing, and variable definitions across diverse cohorts and accelerometer devices. Leading the retrospective harmonisation of demographic, social, behavioural and health-related data. Facilitating prospective harmonisation through questionnaire and protocol development.
Scientific partnerships and events	Managing and expanding existing ProPASS partnerships, including collaborations with external and scientific organisations. Planning and delivering ProPASS events, such as the ProPASS conference, annual symposia and business meetings.
Statistical analysis	Drives methodological innovation and robust statistical analysis for harmonised, multicohort data, supporting both one-stage (individual participant data) and two-stage (traditional meta-analysis) approaches. Enabling reliable and reproducible findings through the development of open-source functions that are specialised for use in federated data analyses.
Scientific outputs	Oversight of ongoing ProPASS projects and publication plans. Aligning research outputs with strategic goals and publication timelines.
ClinPASS	ClinPASS is under development. Focuses on providing resources for research into the secondary and tertiary prevention of non-communicable diseases (eg, cardiovascular disease, cancer, type 2 diabetes or respiratory conditions) by pooling and analysing data across international clinical studies and cohorts. By identifying feasible patterns of physical activity, sleep and posture that reduce recurrent events and improve quality of life, ClinPASS aims to become a unique resource for generating robust, generalisable evidence to inform rehabilitation strategies, refine secondary and tertiary prevention guidelines, and advance our understanding of 24-hour physical behaviours and prognosis in high-risk clinical populations.
Expansion of ProPASS	Identifying new potential ProPASS constituents or collaborators, including cohorts that have completed or are currently undertaking data collection. Providing wearable devices and support for prospective study design, including protocol development, data processing and questionnaire implementation.
PASS*omni* (wearables data processing software)	Dedicated to developing, validating and distributing advanced software for processing thigh- and wrist-worn wearables, enabling precise identification of physical activities, sleep and posture. This open-source tool is critical for ProPASS’s capacity to generate harmonised, high-resolution behavioural data across international cohorts.
Data linkage	Exploring opportunities for data linkage with administrative health records, including hospital admissions, death registries and disease registries. Addressing governance challenges related to access and analysis of these data sources and identifying pathways for effective data harmonisation.
Wearable measurement	Explores, implements and harmonises state-of-the-art wearable technologies—including multidimensional sensors and evolving consumer devices—to improve measurement accuracy and expand the range of detectable behaviours. Underpins ProPASS’s vision for continuous, context-rich health monitoring at scale, integrating both research-grade and consumer wearables.
Federated data infrastructure (DataSHIELD)	Pioneers secure remote data access, and a federated analytic infrastructure—allowing individual-level analyses across international datasets without physically exporting sensitive data outside host jurisdictions. This approach enables the international research community to securely access the longitudinal ProPASS resources to conduct pooled analyses under strict privacy controls, accelerating research while upholding data security and regulatory compliance.

ClinPASS, Clinical Physical Activity, Sedentary Behaviour and Sleep consortium; ProPASS, Prospective Physical Activity, Sitting and Sleep consortium.

In this editorial, we summarise the first phase of ProPASS and its growth in recent years, the first ProPASS scientific outputs,^12 16–18^ key advancements towards unified wearables methodologies, and the ProPASS data resources and how these will be made available to the global research community. To assist future initiatives in this area, we also share the key challenges ProPASS has encountered and discuss mitigation strategies.

## Summary of ProPASS findings to date

All published outputs to date have used the proof-of-concept cross-sectional resource comprising six cohorts from Denmark, Australia, the UK, Finland and the Netherlands.^12 16–18 30^ An additional seven cohorts have subsequently joined the cross-sectional resource from Saudi Arabia, Singapore, South Africa, Spain and China. The published studies have revealed novel insights into physical activity and sleep patterns in relation to cardiometabolic health,^18^ demonstrating that reallocating even small amounts of time from any behaviour into exercise-like activities (eg, running, cycling) was associated with meaningful variations in blood pressure.^16^ This resource also delivered the first pooled analysis exploring physical activity type in relation to cardiometabolic health, showing that approximately 65 min per day of walking and 5 min of stair climbing were each linked to a more favourable cardiometabolic profile, while the adverse effects of sitting became pronounced above 12 hours per day.^17^ In the domain of sleep, ProPASS data have indicated that irregular sleep patterns and lower sleep efficiency (ie, the ratio of total sleep time to time in bed) were associated with increased cardiometabolic risk, independent of sleep duration.^12^ These findings underscore the importance of considering multiple dimensions of sleep health.^30^ Extending this work, the consortium has shown that the combination of irregular or insufficient sleep patterns with lower daily step counts is associated with even higher levels of adiposity, adverse lipid profiles and higher overall cardiometabolic risk.^12^ Expanding on the cross-sectional resource, the main ProPASS longitudinal data resource is currently in the final stages of development. This new resource will enable further exploration of how these behaviours are associated with prospective outcomes such as mortality and noncommunicable diseases. This resource will be remotely accessible to bona fide researchers around the world on application to the ProPASS data access committee.

## ProPASS growth and expansion

### Addressing global evidence inequalities: expansion to low- and middle-income countries and other under-represented countries

To date, the vast majority of physical behaviour evidence, including wearables-based studies, comes from 10% to 15% of the world’s population, that is, high-income European countries, Australia, the USA and Canada; with very limited representation of Asia, Africa or low- and middle-income countries (LMICs).^21^ People in these under-represented parts of the globe exhibit markedly different occupational, transportation and leisure-time patterns of physical activity compared with Western countries, making such research critical for reducing global health inequalities and ensuring representation of all countries in the evidence base underpinning global guidelines. In response to the WHO 2020 Guidelines Development Group’s calls for more evidence from LMICs,^3^ ProPASS has established a formal mechanism to support research teams from under-represented countries to develop wearables-based cohort studies to inform future 24-hour physical activity, sedentary behaviour and sleep guidelines. Such support mechanisms include free loans of wearable devices, research training, protocols and research capacity building for early-career researchers. ProPASS concentrates its LMIC efforts on helping build collaborations, providing access to devices for new studies, and supporting researcher training. As of March 2026, one cohort from South Africa (Middle-Age Soweto Cohort) has joined the consortium, while a ProPASS-initiated studies are planned in Mexico (The Health Workers Cohort), with data collection in progress in Saudi Arabia^31^ and Chile. In addition, pilot data collection is currently being planned to test ProPASS’ wearables methods in the 26-country Prospective Urban Rural Epidemiologic Study.^32^ A major challenge in many LMICs is gaining access to administrative data on morbidity and mortality. ProPASS addresses this challenge through multiple strategies: prioritising cohorts with existing health record linkage infrastructure, supporting applications for linkage with national mortality and disease registries where available and using repeated health assessments during follow-up when administrative linkage is not feasible. However, we acknowledge that outcome ascertainment remains a significant hurdle requiring ongoing methodological development and investment.

### Towards placement-agnostic wearables methods: ProPASS’s methodological foundations

**Figure 1:**
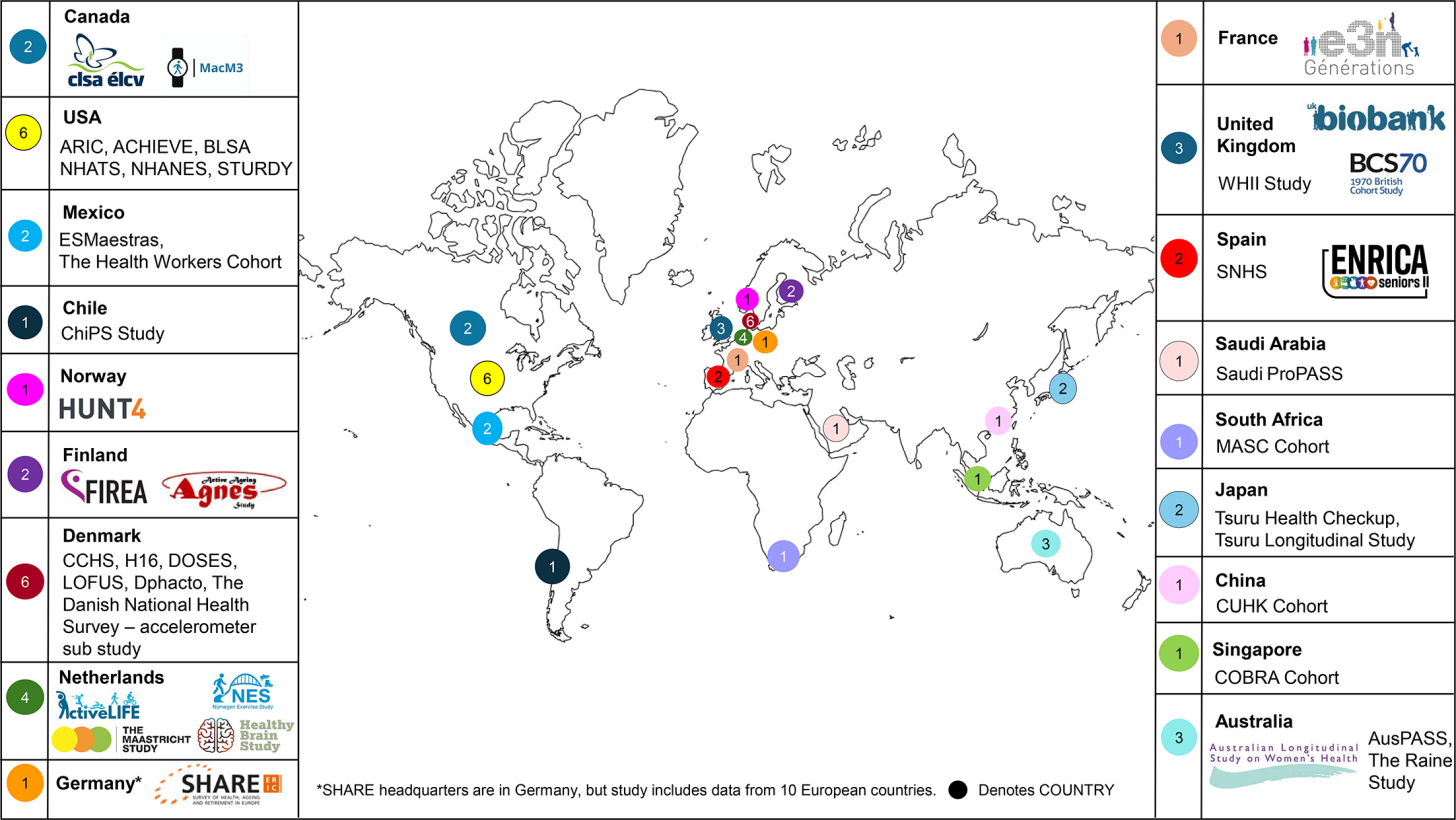
Active ProPASS cohort studies worldwide. The numbers in the circles indicate the total number of cohorts from each country (n=41 total cohorts). CLSA, Canadian Longitudinal Study on Ageing; MacM3, McMaster Monitoring My Mobility Study; ARIC, Atherosclerosis Risk in Communities; ACHIEVE, The Ageing and Cognitive Health Evaluation in Elders; BLSA, Baltimore Longitudinal Study of Ageing; NHATS, National Health and Ageing Trends Study; NHANES, National Health and Examination Survey; STURDY, Rationale and Design of the Study to Understand Fall Reduction and Vitamin D in You; ESMaestras, Mexican Teachers’ Cohort; The Health Workers Cohort; ChiPS, Chilean Health and Physical Activity Study; HUNT-4, The Trøndelag Health Study; FIREA, Finnish Retirement and Ageing Study; AGNES Study, Active ageing—The AGNES Study; CCHS, Copenhagen City Heart Study; H16, Health 2016 Study; DOSES, Danish Observational Study of Eldercare work and musculoskeletal disorders Study; LOFUS, Lolland-Falster Health Study; Dphacto, Danish Physical Activity cohort with Objective measurements; DNHS, The Danish National Health Survey—accelerometer substudy; TMS, The Maastricht Study; NES, Nijmegen Exercise Study; HBS, The Healthy Brain cohort; ActiveLIFE; SHARE, Survey of Health, Ageing and Retirement in Europe; E3N-Generations Cohort Study; WHII Study, Whitehall II Cohort Study; UK Biobank; BCS70, 1970 British Birth Cohort Study; Seniors-Enrica II; SNHS, Spanish National Health Survey; Saudi ProPASS; MASC, The Middle-age Soweto Cohort; Tsuru Health Check-up, Tsuru Longitudinal Study; CUHK, A prospective cohort study of physical activity in Hong Kong; COBRA, Continuous Observations of Behavioural Risk Factors in Asia Study (subcohort of umbrella cohort Singapore Population Health Studies); ALSWH, Australian Longitudinal Study on Women’s Health; AusPASS; The Raine Study.

Currently, ProPASS includes 41 cohort studies (n=2 76 940) from five continents (figure 1)**,** out of which approximately one-third are fully integrated, including legal permission and harmonisation of required data (online supplemental table 1 and 2). Initially set up as a thigh-worn wearables-focused initiative, the consortium’s rapid growth in recent years has been primarily driven by also embracing cohorts featuring wrist-worn wearables, as of 2023. Historically, studies predominantly chose one of three body placements (hip, thigh and wrist), which, until recently, were thought to produce data incompatible with each other, or were limited to a specific brand of devices.^33^ Partly, this separation of methods by device placement was a necessity due to an absence of empirical harmonisation methods for wearables data generated from different placements. ProPASS’ decision to move towards harmonisation of wearable-based methods, which followed the empirical establishment of brand/model-agnostic standards,^20^ was partly motivated by recent landmark papers^7 34–36^ combining data across different wear placements and the rapid growth of machine learning-based wrist accelerometry methods^10 11 37^ that allow high-resolution (eg, 10-second time window) data processing for most posture and activity classes. Using a semisupervised machine learning methodology for optimal data efficiency, ProPASS has developed the first, to our knowledge, machine learning-based measurement schema for thigh-attached wearables; additionally, this schema is tailored to harmonisation of thigh and wrist device data.^30^ The findings of this work are highly encouraging, indicating good to excellent activity type and posture classification precision for both wrist and thigh, and very strong consistency between the two placements. Furthermore, the wrist and thigh harmonised models demonstrated equivalence with ground-truth video direct observation under free-living and semistructured settings for all six activity or posture classes, except for stair climbing, where there is ongoing validation work (online supplemental table 3 and online supplemental figure 2).

## Secondary and tertiary prevention of chronic disease and precision medicine: the ClinPASS consortium

The clinical arm of ProPASS (ClinPASS, currently under development) focuses on the secondary prevention of non-communicable diseases, including cardiovascular disease. By pooling and analysing data across international clinical trials and cohorts, ClinPASS will serve as a unique data resource to inform physical behaviour recommendations aimed at secondary prevention of chronic disease. Specifically, the scientific objectives of ClinPASS include identifying feasible physical behaviour patterns to reduce the risk of recurrent chronic disease events and/or maintain work ability and level of functioning and improve quality of life. Once it reaches maturity, ClinPASS will generate evidence to inform rehabilitation strategies, refine secondary and tertiary prevention guidelines, and advance our understanding of how 24-hour physical behaviours influence prognosis and prevention in high-risk clinical populations.

## ProPASS data resources and data access

As part of its core mission to support the global research community, ProPASS will provide the research community with data resources that are flexible in how they are accessed to maximise cohort participation (see online supplemental figure 3).^38^ The first resource, currently undergoing the last stages of development and testing, is longitudinal and includes ProPASS studies with at least one baseline wearables measurement and either health record linkage or health outcomes available via follow-up data waves. The longitudinal resource can be accessed via two methods: (a) a federated analysis infrastructure based on the DataSHIELD platform,^39^ which enables secure remote individual participant data (IPD) meta-analyses; (b) traditional two-stage meta-analyses, accommodating cohort data that have not joined the DataSHIELD infrastructure. In both longitudinal data resource access scenarios, the datasets stay with the cohorts/data custodians. A second resource involves a pooled cross-sectional resource that originally served as a proof of concept for ProPASS’ data sharing and harmonisation methods.^16–18 20 30 31^ Currently, this resource has six cohorts (approximately n=19 000); with another seven incoming cohorts (approximately n=6000) being fully incorporated by early 2026 (online supplemental table 4). To ensure scientific integrity and high-quality use of the IPD meta-analysis longitudinal data resource, potential users of ProPASS resources will be required to undergo a formal data access application process overseen by the ProPASS Data Access Committee. The data access application process will ensure that proposed research is methodologically and ethically sound, scientific merit is clearly articulated, and there is no potential overlap with existing projects, thereby fostering impactful and collaborative academic research that genuinely advances knowledge (online supplemental figure 1).

## Challenges

Although the first research-grade accelerometers (eg, CSA/WAM 7164 in the mid/late 1990s) predate the first consumer-grade devices (Polar FA20, 2007; Fitbit Classic, 2009), there has been relatively little technological advancement with research-grade hardware over the last decade. As of 2025, the typical research-grade wearable suitable for large-scale cohort studies features dated (>15-year-old) technology (ie, a single three-axial accelerometry sensor), limiting the capacity of current ProPASS studies to capture complete physical behaviours, such as activity/exercise type, strength-demanding activities (lifting, carrying, pushing and pulling), sleep behaviours and walking on a gradient, as well as cardiorespiratory intensity of the activity. All these could be enabled by the addition of inexpensive standard sensors such as photoplethysmography (PPG) for heart rate indicating cardiorespiratory intensity, gyroscope for detecting orientation and rotational movement, and barometer (for altitude), and expansion of algorithmic development beyond intensity and bout predictions. While multisensor research-grade devices (incorporating, eg, gyroscopes, PPG sensors and altimeters) are available, they are not widely used in large-scale epidemiological cohort studies. However, it is likely that their adoption will increase in the future.

The bespoke ProPASS federated infrastructure (DataSHIELD) for secure remote pseudo-IPD access/analyses from anywhere around the world will be the state-of-the-art methodology for data sharing in epidemiological research. Although DataSHIELD is widely recognised for enabling non-disclosive federated analysis, the integration of advanced methods like compositional data analysis (CoDA)^16^ has necessitated substantial financial commitment and technical management outsourcing. ProPASS is at the forefront of extending DataSHIELD’s capabilities, pioneering the large-scale implementation of CoDA for device-measured physical activity epidemiology on a global scale. The relatively recent General Data Protection Regulation (GDPR) legislation has increased the need for compliance awareness and has made information technology teams in some institutions cautious, requesting multiple safeguards and administrative procedures that may not be realistic or feasible. The data sharing regulations in some countries restrict analyses of cohort data involving linked administrative health records (eg, mortality, hospital admissions and cancer registrations) in a closed ecosystem, limiting federated access via DataSHIELD.

Despite significant resources invested in the expansion to LMICs and other under-represented countries, and the substantial material support offered by the consortium (eg, offering free wearable device loans), to date only three studies have been completed or are in progress. In many cases, the economic and political reality has hindered discussions with research teams from, for example, Africa and South America, where securing basic resources to complete the fieldwork is often an insurmountable challenge. To overcome these barriers, ProPASS provides equipment and long-term capacity building, including the training necessary for data collection. Another challenge for ProPASS is securing long-term, stable funding to support sustainable growth, as the consortium currently relies on competitive, priority-driven, national funding mechanisms for its ongoing development and expansion. This necessitates ongoing peer review and scientific assessment to ensure that ProPASS continues to make a meaningful contribution as the resource matures.

## Future plans and opportunities

Looking ahead, the future of wearables for physical activity, sedentary behaviour and sleep assessment within ProPASS is poised for a major leap, moving beyond the current reliance on a single triaxial accelerometer. Progress in multisensor hardware, combining accelerometry with heart rate and even biosignal measurements (eg, non-invasive blood glucose and blood pressure monitoring), is paving the way for richer, more precise detection of activity types, postures and sleep stages, overcoming previous stagnation in sensor application. At the same time, consumer wearables are rapidly advancing, with devices such as smartwatches, rings and next-generation trackers now used for key sleep and activity parameters and capable of large-scale, real-world data collection.^40 41^ ProPASS’ plans include integrating studies that use an individual’s own consumer-grade wearable data (eg, the All of Us cohort in the USA,^42^ a particularly exciting development that could enable prolonged periods of data capture (months/years) and data enrichment from multiple sensors. However, consumer wearables present methodological challenges that must be carefully addressed. These include proprietary algorithms with limited transparency, variable accuracy across devices and brands, frequent firmware updates that can alter measurement characteristics, limited access to raw sensor data, and a lack of standardisation in data outputs across manufacturers.^43^ Despite these challenges, consumer wearables represent a valuable frontier for capturing real-world behavioural patterns at scale, reaching more diverse populations with longer assessment periods. Integrating such multisensor systems could expand measurement to capture continuous and context-rich behaviour data. When these developments are complete, the ProPASS data resources will not only provide insight into physical behaviour quantity, but also physical activity and sleep physiology, posture, stress, and chronic disease risk in unprecedented detail, supporting precision public health and translational research across populations (see online supplemental table 5).

## Conclusion

ProPASS’s foundational vision was to shape future guidelines, interventions and precision medicine initiatives aimed at supporting movement and sleep behaviour change for primary and secondary chronic disease prevention and disease management across the globe. In this extended editorial, we provide an update on the consortium’s current and future directions, how it is contributing to the evolution of the broader physical activity field, and have highlighted some of the key challenges. In summary, despite the substantial progress ProPASS and the broader field have achieved in recent times, there is significant ground to cover in the next 5–10 years. Importantly, the ProPASS experience to date highlights once more the power of collaborative synergies and the value of promoting universal standards in science.

## Supplementary material

10.1136/bjsports-2025-111283 online supplemental file 1
