# Supplementary material for: Prospective Physical Activity, Sitting and Sleep consortium (ProPASS): addressing methodological and geographical barriers to inform global public health guidelines, interventions and precision medicine
Source: Br J Sports Med. 2026 Jun 22;60(16):e111283. doi: 10.1136/bjsports-2025-111283 (PMC13479634; doi:10.1136/bjsports-2025-111283)
Supplement: Supplementary file 1 [file bjsports-60-16-s001.pdf]

Supplementary Document

Supplementary Figure 1: Overview of the ProPASS Governance Structure.

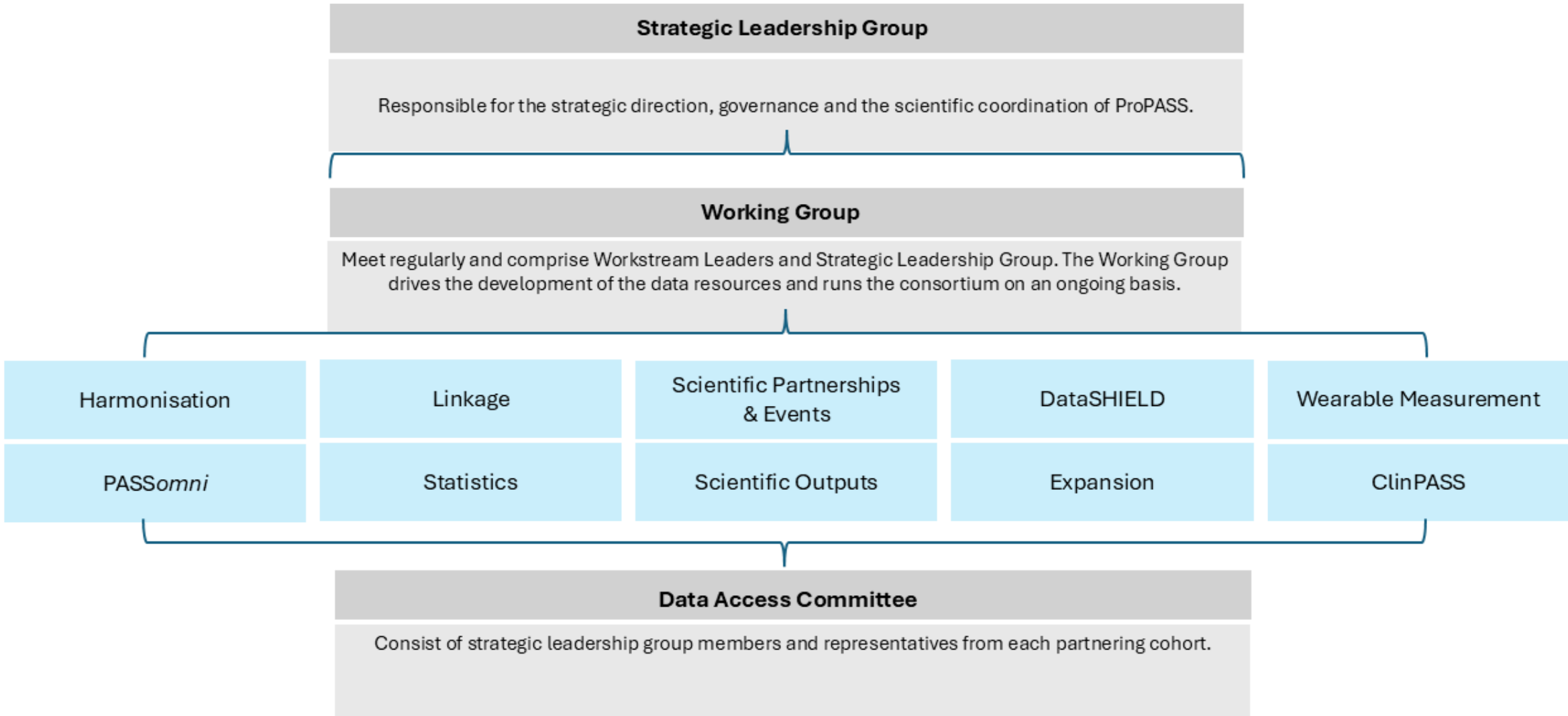

Supplementary Figure 2: Equivalence testing of thigh and wrist to ground-truth. Positive values indicate overestimation and negative values indicate underestimation.

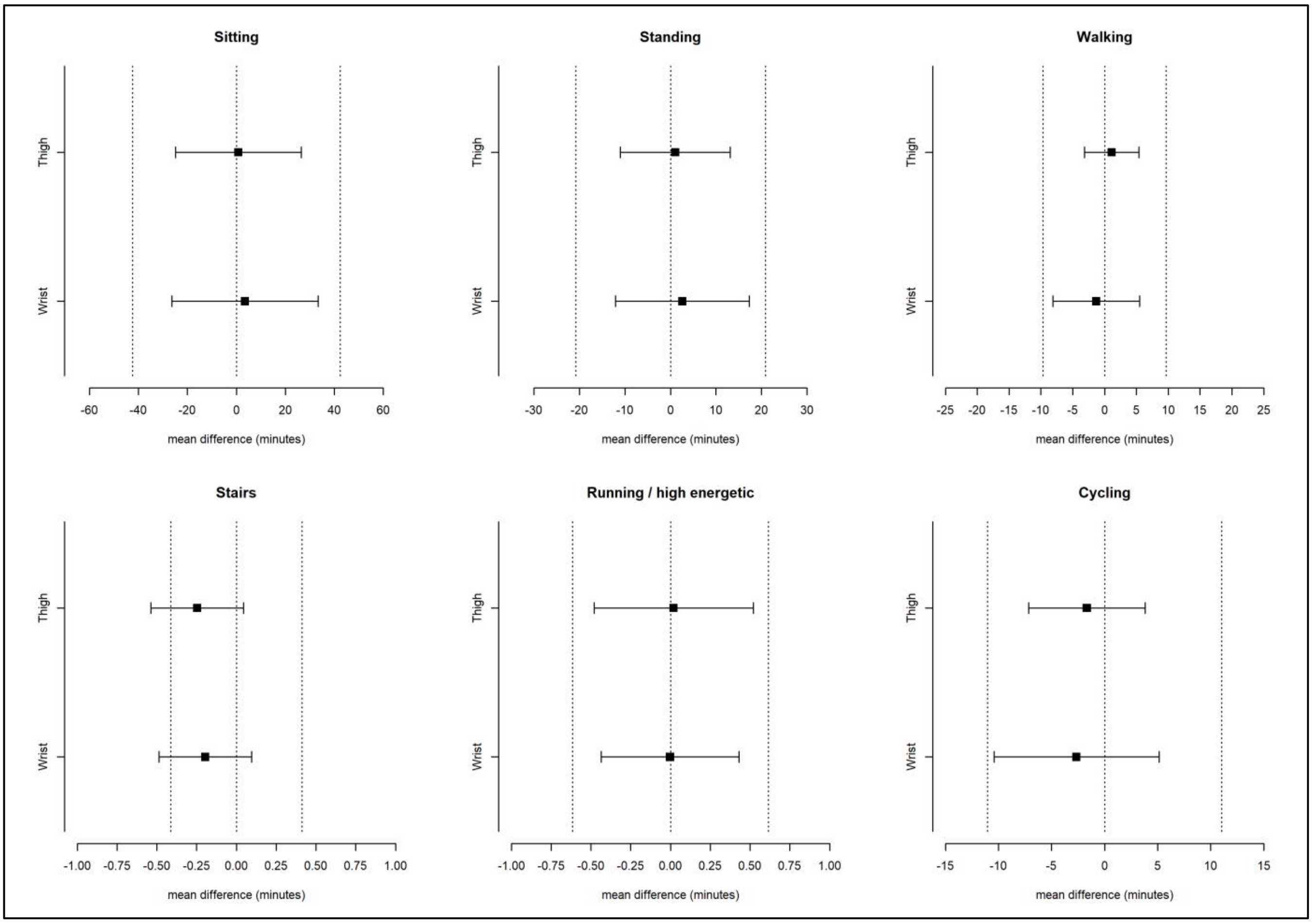

The upper and lower bounds reflect a Cohen's d of 0.3, representing a small-to-medium effect size. Equivalence was defined as being within Cohen's d of 0.3. Figure reprinted from Ahmadi et al. (2026)[1].

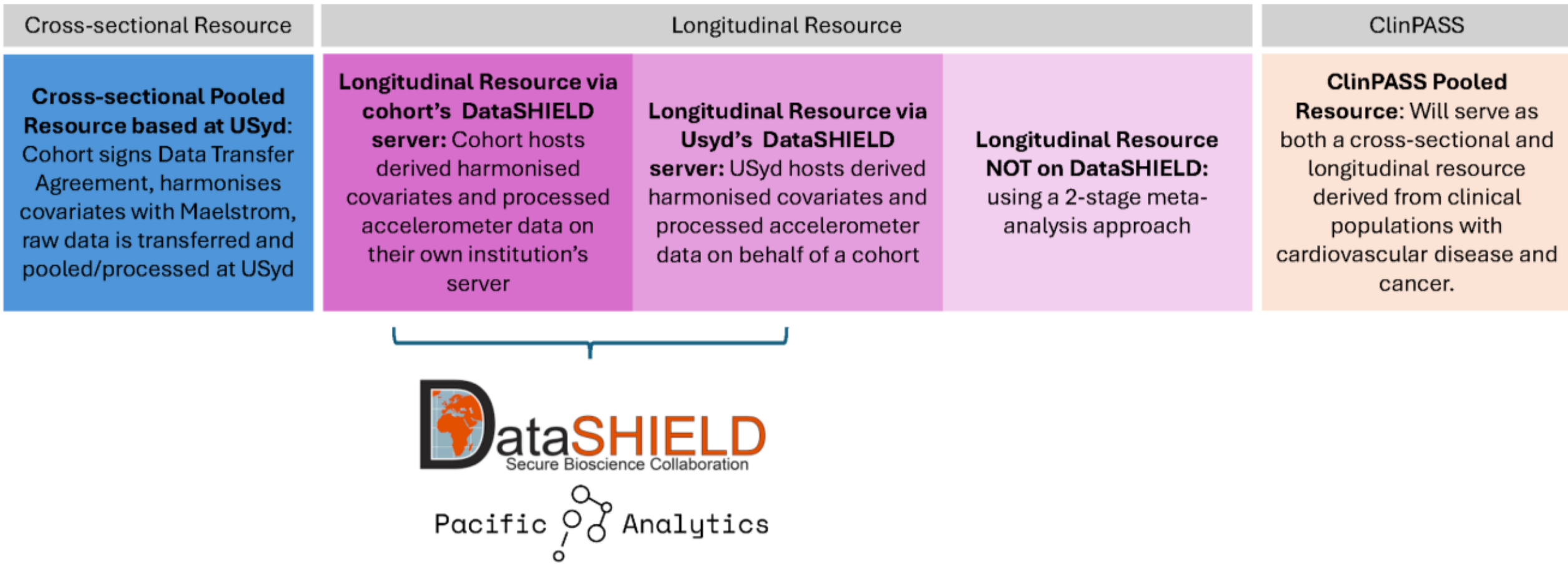

Supplementary Figure 3: Overview of ProPASS's data resources.
The cross-sectional resource comprises centrally located (pooled) cohort data with risk factors as outcomes. The longitudinal resource comprises prospective cohort data that can be accessed and analysed remotely via the DataSHIELD platform, or using a traditional two-stage meta-analytic approach, when participation in DataSHIELD is not feasible. The ClinPASS resources will contribute both cross-sectional and longitudinal data from clinical populations.

Supplemental Table 1. Retrospective accelerometry studies (n=32) supporting the Prospective Physical Activity, Sitting, and Sleep consortium (ProPASS).

Main Study Name / Country	Leading Institution	Population / Age Range	Device	Device Placement	n with Accelerometry Data	Wearables Data Collection
The Raine Study (Australia)	Curtin University	General population / mean age Generation 1: 55 years; mean age Generation 2: 22 years	ActiGraph (Wrist) and SENS motion (Thigh)	Wrist and Thigh	~3,000**	Wrist: 2010-2018 Thigh: 2023-2025
ALSWH: Australian Longitudinal Study of Women's Health (Australia)	The University of Queensland and The University of Sydney	General population / 45-50 years	ActivPAL3 and ActivPAL4 micro	Thigh	~1,000	2019-2020
Active Ageing- The AGNES Study (Finland)	University of Jyväskylä	General Population / 75-85 years	UKK RM42	Thigh	~300	2017-2018, 2021-2022
FIREA: Finnish Retirement and Aging Study (Finland)	University of Turku	General Population / Occupational cohort / 58-64 years at baseline	ActiGraph wActiSleep-BT and wGT3X-BT (Wrist) Axivity AX3 (Thigh)	Wrist and Thigh	~1,200 comprising of: 900 wrist only 300 thigh and wrist	Wrist: 2014-2025 Thigh: 2015-2025
UK Biobank Study (UK)	University of Oxford	General population / 40-69 years	Axivity AX3	Wrist	~100,000	2013-2015
WHII: Whitehall II Cohort Study	University College London	General population / 60-83 years	GENEActiv	Wrist	~4,000	2012-2013

(UK)						
BCS70: 1970 British Birth Cohort Study<br><br>(UK)	University College London	General population / 47-49 years	ActivPAL3 micro	Thigh	~5,500	2016-2018
CCHS: Copenhagen City Heart Study<br><br>(Denmark)	Frederiksberg Hospital, Copenhagen	General Population /18+ years	Actigraph GT3X	Thigh	~1,700	2011-2015
Dphacto: Danish PHysical ACTivity cohort with Objective measurements Study<br><br>(Denmark)	National Research Centre for the Working Environment, Copenhagen	Workers in cleaning, manufacturing, and transportation companies / 18-67 years	Actigraph GT3X	Thigh	~800	2012-2014
DOSES: Danish Observational Study of Eldercare work and musculoskeletal disorderS Study<br><br>(Denmark)	National Research Centre for the Working Environment, Copenhagen	Eldercare workers / 18-67 years	Actigraph GT3X	Thigh	~500	2013-2014
Health 2016 Study<br><br>(Denmark)	Centre for Clinical Research and Prevention, Frederiksberg	General Population / 18-69 years	Axivity	Thigh	~800	2016-2017
The Danish National Health Survey – accelerometer substudy	The National Institute of Public Health, University of Southern Denmark	General population / 16 years or older	SENS Motion	Thigh	1,050	2023

(Denmark)						
LOFUS: Lolland-Falster Health Study<br><br>(Denmark)	Zealand University Hospital, Nykøbing Falster	General Population / (18+ years eligible)	Axivity 3	Thigh	~4,000	2017-2020
HUNT 4: Trøndelag Health Study<br><br>(Norway)	Norwegian University of Science and Technology	General Population / 18 years or older	Axivity AX3	Thigh and lower back	~28,000	2017-2019
NES: Nijmegen Exercise Study<br><br>(Netherlands)	Radboud university medical centre	Participants in Nijmegen 4-day Marches or Seven Hills Run, and their friends & family members / 18+ years	ActivPAL3	Thigh	~2,500	2021-2026
The Maastricht Study<br><br>(Netherlands)	Maastricht University	General Population (Oversampling of those with T2 Diabetes) / 40-75 years	ActivPAL3	Thigh	~9,000	2010-2019
The Healthy Brain Cohort<br><br>(Netherlands)	Radboud university medical centre	30-40 years	ActivPAL3	Thigh	731	2019-2023
ActiveLIFE<br><br>Real-world physical ACtivity assessmenT to develop personalized physIcal actiVity and sEdentary	Radboud University Medical Center	General population / 12 - 95 years	Axivity AX3	Thigh and Thigh + wrist in a subsample	~60,000**	2025 – 2029***

behavior recommendations in healthy and diseased individuals: a LIFElines Biobank study<br><br>(Netherlands)						
COBRA: Continuous Observations of Behavioural Risk Factors in Asia study<br><br>(Singapore)	National University of Singapore	General Population / 30-69 years	Axivity 3	Thigh	~1,000	2021-2024
SHARE: Survey of health, aging and Retirement in Europe<br><br>(Belgium, Czech Republic, Denmark, France, Germany, Italy, Poland, Spain, Slovenia, and Sweden)	Max Planck Institute, Berlin	General Population / 50+ years	Axivity 3	Thigh	~900	2019-2020
MASC: Middle-age Soweto Cohort<br><br>(South Africa)	University of the Witwatersrand	General Population / 50+ years	ActivPAL3	Thigh	~900	2017-2018
SENIORS-ENRICA 2<br><br>(Spain)	Universidad Autónoma de Madrid	Older people (≥65years at baseline)	Axivity AX3	Thigh	~950 (3rd wave)	Thigh: 2021-2023 (3rd wave – the first one with thigh acc.)

CLSA: Canadian Longitudinal Study on Aging<br><br>(Canada)	McMaster University	Community-dwelling sample of older adults aged >=55 years at the time of accelerometer data collection	TicWatch Pro 3 Ultra GPS (Wrist)<br><br>ActiGraph wGT3X-BT (Thigh)	Thigh and Wrist	~10,000	2021-2024
MacM3: McMaster Monitoring My Mobility study<br><br>(Canada)	McMaster University	Community-dwelling older adults 65 years and older	TicWatch Pro 3 Ultra GPS<br><br>ActiGraph wGT3X-BT<br><br>Axivity ax3	TicWatch: Wrist<br><br>ActiGraph/Axivity: Thigh	TicWatch: 1,475<br><br>Actigraph: 1,007<br><br>Axivity:150	2022-2024
NHATS: National Health and Aging Trends Study<br><br>(USA)	Johns Hopkins University	65+ years	Actigraph Centrepoint watch	Wrist	1,000	2021 – present
STURDY: Rationale and design of the Study To Understand Fall Reduction and Vitamin D in You<br><br>(USA)	Johns Hopkins University	70+ years	ActigraphGT9x	Wrist	688	2015-2018
BLSA: Baltimore Longitudinal Study of Aging<br><br>(USA)	National Institutes of Health (NIH) / National Institute on Aging (NIA)	General population (initially healthy adults) / 22-97 years	Actigraph GT9x	Wrist	939	2015 - present

ARIC: Atherosclerosis Risk in Communities<br>(USA)	Johns Hopkins University	70+ years	ActigraphGT9x	Wrist	1,250	2020-2022
ACHIEVE: The Aging and Cognitive Health Evaluation in Elders<br>(USA)	Johns Hopkins University	70+ years	Actigraph<br>GT9x	Wrist	850	2017-2019
NHANES: National Health and Nutrition Examination Survey<br>(USA)	National Center for Health Statistics	General population / 20-80 years	ActiGraph model GT3X+	Wrist	~7,000	2011-2014
Tsuru Longitudinal Study<br>(Japan)	Kanagawa University of Human Services	General population / 65 years and older	wGT3X-BT	Wrist	~1,000**	2024-2026***
Tsuru Health Checkup (Japan)	Kanagawa University of Human Services	General population /<br>75-82 years	wGT3X-BT	Wrist	~500**	2022-2026***

** anticipated sample size; *** anticipated completion of baseline data collection.

Supplemental Table 2. Prospective accelerometer studies (n = 9) supported by the Prospective Physical Activity, Sitting, and Sleep consortium (ProPASS), joining ProPASS upon completed data collection.

Main Study Name / Country	Leading Institution	Population / Age Range	Device	Device Placement	n with Accelerometry Data*	Baseline (accelerometry collection)**
Spanish National Health Survey (Spain)	Universidad Europea de Madrid	General population / 30-75 years	SENS motion	Thigh	~4,000	2027-2028
CUHK: A prospective cohort study of physical activity in Hong Kong (Hong Kong, China)	The Chinese University of Hong Kong	Community-dwelling older adults - 65 years or above	ActivPAL3 and ActivPAL4 micro	Thigh	~700	2022-2023
Saudi ProPASS (Saudi Arabia)	King Faisal University	General population / >30 years	ActivPAL3 and ActivPAL4 micro	Thigh	~900	2023-2025
Chilean Health and Physical Activity Study – ChiPS (Chile)	Universidad de O'Higgins	General population / 18-65 years	ActivePal3 ActivPAL4 micro	Thigh	~550	2024-2025
E3N-Generations pilot (France)	National Institute of Health & Medical Research (Inserm)	General Population / ~50 years	Axivity AX3	Thigh and Wrist	~100-150	2026
E3N- Generations main study (France)	National Institute of Health & Medical Research (Inserm)	General Population / ~50 years	Axivity AX3	Thigh and/or Wrist dependent on pilot outcomes	~5,000-7,000	2027-2028

Mexican Teachers' Cohort (ESMaestras)<br><br>(Mexico)	National Institute of Public Health (INSP)	55+ years	Axivity and ActivPAL	Wrist with a sub-sample of thigh & wrist	~3000	2025-2026
The Health Workers Cohort<br><br>(Mexico)	National Autonomous University of Mexico (UNAM)<br><br>National Institute of Public Health (INSP)<br><br>Mexican Social Security Institute (IMSS)	Health professionals and family members /<br><br>20-90 years	Axivity and ActivPAL	Wrist with a sub-sample of thigh & wrist	~2,500-3,000	2025-2026
AusPASS<br><br>(Australia)	University of Sydney	Older population /<br><br>65+ years	Axivity	Thigh and wrist	10,000	2025-2027

*anticipated sample size; **anticipated data collection timeframe.

Supplementary Table 3: Physical activity and posture duration (minutes) per participant in free-living evaluation

Placement/ground-truth	Sit	Stand	Walk	Stairs	Run/energetic activity	Cycle
**Wrist**	197.6 ± 57.3	109.2 ± 53.4	36.6 ± 23.9	3.7 ± 1.0	2.8 ± 1.5	9.0 ± 27.7
**Thigh**	194.8 ± 57.1	107.7 ± 52.0	40.2 ± 24.3	4.2 ± 0.5	3.1 ± 0.3	9.6 ± 31.0
**Video direct observation**	194.0 ± 54.8	106.6 ± 50.8	39.1 ± 24.4	4.0 ± 1.0	3.0 ± 1.5	12.2 ± 27.5
**Absolute SD[a]**	6.1 ± 6.1	11.0 ± 9.0	2.3 ± 2.2	0.2 ± 0.1	0.4 ± 0.2	1.6 ± 4.5
**CV[b]**	.078	.140	.063	.041	.022	.083

Physical activity and posture classifications are based on the self-trained semi-supervised wrist and thigh classifiers and the video direct observation ground-truth labels from the free-living evaluation.
N=53; Females = 35; Mean (SD) age= 55.4 (13.1) years
[a]Absolute SD reflects absolute disagreement between wrist and thigh on a participant-by-participant basis. It is calculated as the average standard deviation in activity and posture durations between the wrist and thigh for each participant
[b]CV=coefficient of variation. σ/A; where σ = SD and A = the mean activity and posture duration between the wrist and thigh classifiers for each participant
Reprinted from Ahmadi et al (2026)[1].

Supplemental Table 4: Existing and incoming cohorts joining ProPASS's cross-sectional resource.

Existing cohorts		
**Cohort**	**Host institution / Country**	**(n)**
**ALSWH**: Australian Longitudinal Study on Women's Health	University of Queensland / Australia	1,000
**BCS70**: 1970 British Birth Cohort Study	University College of London / Great Britain	5,500
**DPhacto**: Danish Physical Activity cohort with Objective measurements	National Research Centre for the Working Environment, Copenhagen / Denmark	800
**TMS**: The Maastricht Study	Maastricht University / Netherlands	9,000
**NES**: Nijmegen Exercise Study	Radboud university medical centre / Netherlands	2,000
**FIREA**: Finnish Retirement and Aging Study	University of Turku / Finland	1,200
		N=19,000
**Incoming cohorts**		
**Cohort**	**Host institution / Country**	**(n)**
**COBRA**: Continuous Observations of Behavioural Risk Factors in Asia study	National University of Singapore / Singapore	1,000
**DOSES**: Danish Observational Study of Eldercare work and musculoskeletal disorders study	National Research Centre for the Working Environment, Copenhagen / Denmark	500
**MASC**: The Middle-age Soweto Cohort	University of the Witwatersrand / South Africa	900
**ERC**: SENIORS-ENRICA 2	Universidad Autónoma de Madrid / Spain	975
**CUHK**: A prospective cohort study of physical activity in Hong Kong	The Chinese University of Hong Kong / Hong Kong, China	700
**HBS**: Healthy Brain cohort	Radboud university medical centre / Netherlands	731
**KSA**: Saudi ProPASS	King Faisal University / Saudi Arabia	900
		N=5,681

*Total pooled sample size between existing and incoming cohorts (N) = 24,681.

Supplemental Table 5. ProPASS Key Challenges and ProPASS Mitigations.

Key Challenges	ProPASS Mitigation Plans
Incomplete accounts of physical activity behaviours due to limited sensors within standard research-grade devices	Exploring future use of sensor-rich consumer-grade wearable devices in cohort studies in partnership with the International Society for the Measurement of Physical Behaviour.
Federated health data linkage and access limitations for large-scale cohort analyses	Develop an external 2-stage meta-analysis infrastructure/
Political and economic instability within LMICs limit expansion to under-represented countries	Partner directly with existing LMIC research institutions to create sustainable, long-term collaborations - with a focus on building their resources and research capacity in partnership with the International Society of Physical Activity and Health

References

1. Ahmadi MN, Koemel N, Biswas R, et al. Development and evaluation of wrist- and thigh-worn accelerometer algorithms using self-training machine learning models for classification of activity type and posture: towards device placement-agnostic methods in the ProPASS consortium. *International Journal of Behavioral Nutrition and Physical Activity*. 2026;doi: 10.1186/s12966-026-01930-z
